# Cyclic di-GMP-dependent Signaling Pathways in the Pathogenic Firmicute *Listeria monocytogenes*

**DOI:** 10.1371/journal.ppat.1004301

**Published:** 2014-08-07

**Authors:** Li-Hong Chen, Volkan K. Köseoğlu, Zehra T. Güvener, Tanya Myers-Morales, Joseph M. Reed, Sarah E. F. D'Orazio, Kurt W. Miller, Mark Gomelsky

**Affiliations:** 1 Department of Molecular Biology, University of Wyoming, Laramie, Wyoming, United States of America; 2 Department of Microbiology, Immunology, and Molecular Genetics, University of Kentucky, Lexington, Kentucky, United States of America; University of Michigan Medical School, United States of America

## Abstract

We characterized key components and major targets of the c-di-GMP signaling pathways in the foodborne pathogen *Listeria monocytogenes*, identified a new c-di-GMP-inducible exopolysaccharide responsible for motility inhibition, cell aggregation, and enhanced tolerance to disinfectants and desiccation, and provided first insights into the role of c-di-GMP signaling in listerial virulence. Genome-wide genetic and biochemical analyses of c-di-GMP signaling pathways revealed that *L. monocytogenes* has three GGDEF domain proteins, DgcA (Lmo1911), DgcB (Lmo1912) and DgcC (Lmo2174), that possess diguanylate cyclase activity, and three EAL domain proteins, PdeB (Lmo0131), PdeC (Lmo1914) and PdeD (Lmo0111), that possess c-di-GMP phosphodiesterase activity. Deletion of all phosphodiesterase genes (*ΔpdeB/C/D*) or expression of a heterologous diguanylate cyclase stimulated production of a previously unknown exopolysaccharide. The synthesis of this exopolysaccharide was attributed to the *pssA-E* (*lmo0527-0531*) gene cluster. The last gene of the cluster encodes the fourth listerial GGDEF domain protein, PssE, that functions as an I-site c-di-GMP receptor essential for exopolysaccharide synthesis. The c-di-GMP-inducible exopolysaccharide causes cell aggregation in minimal medium and impairs bacterial migration in semi-solid agar, however, it does not promote biofilm formation on abiotic surfaces. The exopolysaccharide also greatly enhances bacterial tolerance to commonly used disinfectants as well as desiccation, which may contribute to survival of *L. monocytogenes* on contaminated food products and in food-processing facilities. The exopolysaccharide and another, as yet unknown c-di-GMP-dependent target, drastically decrease listerial invasiveness in enterocytes in vitro, and lower pathogen load in the liver and gallbladder of mice infected via an oral route, which suggests that elevated c-di-GMP levels play an overall negative role in listerial virulence.

## Author Summary

*Listeria monocytogenes* is ubiquitously present in the environment, highly adaptable and tolerant to various stresses. *L. monocytogenes* is also a foodborne pathogen associated with the largest foodborne outbreaks in recent US history. Signaling pathways involving the second messenger c-di-GMP play important roles in increased stress survival of proteobacteria and mycobacteria, yet roles of c-di-GMP signaling pathways in *L. monocytogenes* have remained unexplored. Here, we identified and systematically characterized functions of the proteins involved in c-di-GMP synthesis, degradation and sensing. We show that elevated c-di-GMP levels in *L. monocytogenes* result in synthesis of a previously unknown exopolysaccharide that promotes cell aggregation, inhibits motility in semi-solid media, and importantly, enhances bacterial tolerance to commonly used disinfectants as well as desiccation. These properties of the exopolysaccharide may increase listerial survival in food processing plants as well as on produce during transportation and storage. Elevated c-di-GMP levels also grossly diminish listerial invasiveness in enterocytes in vitro, and impair bacterial accumulation in selected mouse organs during oral infection.

## Introduction

Cyclic dimeric GMP (c-di-GMP) [1] is one of the most common bacterial second messengers. Over the last ten years our understanding of c-di-GMP-mediated signal transduction pathways has rapidly expanded (reviewed in [2]–[6]). However, this expansion has been dominated by studies of Proteobacteria, and to a lesser extent Actinobacteria and Spirochetes, while studies of c-di-GMP signaling in Firmicutes have lagged behind. In the Proteobacteria, elevated levels of intracellular c-di-GMP are associated with inhibition of motility and increased synthesis of biofilm components, e.g. exopolysaccharides (EPS), pili and/or surface adhesins. In pathogens that propagate extracellularly, elevated c-di-GMP levels have been found generally detrimental for acute infections (reviewed in [2], [5]–[7]), although individual components of c-di-GMP signaling networks may play different roles during various stages of infection [8], [9]. In contrast, during chronic infections, c-di-GMP-induced biofilms greatly increase pathogen survival in vivo [10]–[12]. In intracellular proteobacterial pathogens, c-di-GMP signaling pathways are required for full-scale virulence in those species that form biofilm-like intracellular structures [13]–[15], but appear to be detrimental, at least in some species, that do not form such structures [16].

In this study, we used the foodborne pathogen *Listeria monocytogenes* [17] as a model to gain insight into c-di-GMP-based regulation in Firmicutes in general. *L. monocytogenes* is widespread in the environment. It has been isolated from soil, silage, groundwater, sewage and vegetation and actively grows at a broad range of temperatures (from 0 to 44°C), oxygen levels, pH (from 4.4 to 9.6), and salt concentrations (up to 10% w/v NaCl), and is capable of utilizing a variety of carbohydrates as well as other organic molecules as carbon sources. Listeriosis is a relatively infrequent disease but it has the highest mortality rate, ∼20%, among foodborne diseases in the developed world. The complications of listeriosis, common in immunocompromised patients, include encephalitis, meningitis, and stillbirths or infection of the central nervous system in newborns [17]–[20].

Common sources of listerial contamination include unpasteurized milk and milk products, raw meat, and packaged cooked meat products. In plants processing meat and milk products listerial biofilms can persist for years and even decades and cause repetitive contamination of processed foods [21], [22]. In recent years, listeriosis caused by contaminated fresh produce has become a significant concern. According to The Centers for Disease Control and Prevention, the 2011 outbreak caused by *Listeria*-contaminated cantaloupes resulted in 33 deaths and was the largest foodborne disease outbreak in US history in almost 90 years [23], [24]. Our understanding of how listeria attach and grow on the surfaces of produce is surprisingly poor, and so is our knowledge of the mechanisms ensuring long-term listerial survival. EPS is one of the common components that facilitate bacterial attachment to plant surfaces and increases their tolerance to desiccation and disinfection [25], [26], both of which are critical parameters for food safety. However, the ability of *L. monocytogenes* to synthesize EPS has remained controversial [27], [28].

In Proteobacteria, EPS synthesis is commonly induced via c-di-GMP signaling pathways, yet studies of such pathways in Firmicutes are just beginning to emerge [29]–[32]. It is peculiar that distribution of c-di-GMP signaling pathways in Firmicutes is very uneven. Several major genera of pathogenic firmicutes, *Staphylococci*, *Streptococci* and *Enterococci*, lack these altogether. However, staphylococci retain remnants of c-di-GMP signaling enzymes, which are involved in biofilm regulation but are no longer associated with c-di-GMP [33]. On the other extreme of the spectrum are certain clostridial species, e.g. *Clostridium difficile*, that have numerous enzymes involved in c-di-GMP synthesis and hydrolysis [29]. A recent study by Tamayo and colleagues [30] showed that elevated levels of c-di-GMP inhibited motility and induced cell aggregation in *C. difficile*. The c-di-GMP-dependent riboswitches from *C. difficile* expressed in a heterologous host were shown to affect gene expression in a c-di-GMP-dependent manner. One riboswitch is located upstream of the *C. difficile* flagellar biosynthesis operon [34]; the other one is part of the riboswitch-ribozyme system predicted to control adhesin gene expression [35]. Other recent studies [31], [32] characterized enzymes involved in c-di-GMP synthesis and degradation in *Bacillus subtilis* and uncovered the role of c-di-GMP in regulating motility and biofilm formation in this species.

Here, we present a genome-wide view of c-di-GMP signaling in *L. monocytogenes*. We used bioinformatics analysis to identify genes involved in c-di-GMP synthesis, degradation and signal transduction, and subsequently applied genetic and biochemical approaches to characterize functions of these genes in EPS synthesis, motility inhibition, tolerance to disinfection and desiccation, invasiveness in mammalian cells, and virulence in a mouse model of listeriosis.

## Results

### Bioinformatic analysis of the c-di-GMP signaling system in *Listeria*

**Figure 1:**
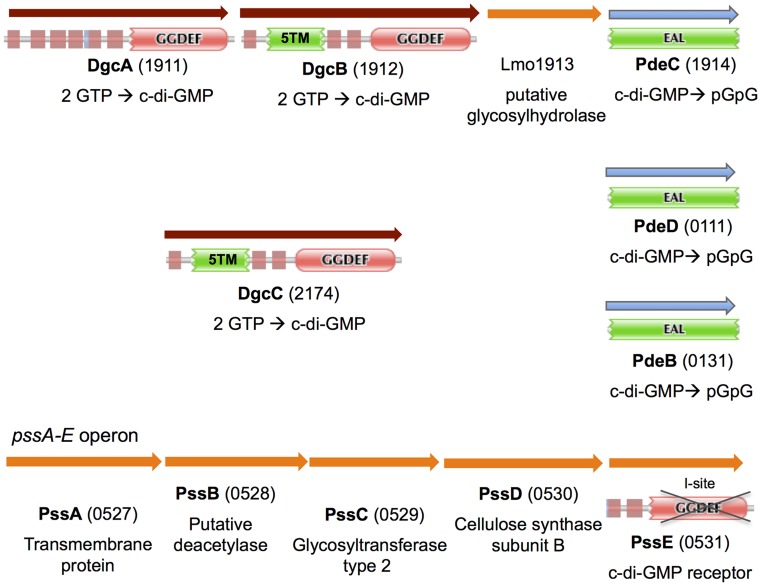
In silico analysis of genes and proteins involved in c-di-GMP signaling in *L. monocytogenes*. Depicted are genes predicted to encode DGCs (DgcA-C), c-di-GMP PDEs (PdeB-D), a c-di-GMP receptor (PssE), and listerial EPS biosynthesis machinery. Protein domain architectures are taken from the Pfam database: 5TM, a conserved five-transmembrane module; unmarked red box, transmembrane domain; crossed GGDEF domain, enzymatically inactive GGDEF domain.

C-di-GMP is synthesized by diguanylate cyclases (DGCs), which contain GGDEF domains [36], [37], and degraded by c-di-GMP-specific phosphodiesterases (PDEs), which contain either EAL [38]–[40] or HD-GYP [41] catalytic domains. The currently sequenced strains of *L. monocytogenes*, and the majority of related listerial species encode four GGDEF domain proteins, three EAL domain proteins and no HD-GYP domain proteins (Pfam database [42]) (Fig. 1).

Our sequence analysis predicted that three of the four GGDEF proteins from *L. monocytogenes* EGD-e, Lmo1911 (DgcA), Lmo1912 (DgcB) and Lmo2174 (DgcC), contain conserved residues associated with DGC activity, and therefore they likely possess DGC activities [4], [43]. The three predicted DGCs have similar domain architectures with a GGDEF domain preceded by either six or eight transmembrane helices (Fig. 1). This domain architecture suggests that c-di-GMP synthesis is regulated by external signals or signals derived from the cell wall or cytoplasmic membrane. The three proteins share approximately 30% identity to each other over their entire lengths, and may have resulted from ancient gene duplications. The EAL domain proteins in strain EGD-e, Lmo0131 (PdeB), Lmo1914 (PdeC) and Lmo0111 (PdeD), have conserved residues required for c-di-GMP binding and hydrolysis [4], [44], and therefore were expected to possess PDE activities (Fig. 1). These putative PDEs contain only single EAL domains suggesting their cytoplasmic localization.

The *dgcA* and *dgcB* genes are codirectional and separated from each other by 20 bp, which indicates that they likely form an operon. The *pdeC* gene appears to belong to the same *dgcA-dgcB-lmo1913-pdeC* (*lmo1911-1914*) operon. Tiling microarray expression data support an operonal structure of this gene cluster [45]. The intervening gene, *lmo1913*, encodes a protein of unknown function. Based on structural predictions, Lmo1913 belongs to the six-hairpin glycosidase superfamily [46] (Fig. 1). Therefore, DgcA, DgcB and PdeC may represent a signaling module involved in c-di-GMP synthesis and degradation, and this module may be involved in controlling synthesis of an unknown EPS.

The GGDEF domain of the fourth GGDEF protein, Lmo0531, is clearly degenerate. The signature GG(D/E)EF motif in Lmo531 is ^208^DKDDA, which should make this protein incapable of c-di-GMP synthesis (Fig. 1). Five amino acids upstream of the signature motif is an RxxD motif that represents a part of a c-di-GMP-binding sequence known as an I-site [43], [47]. Therefore, we hypothesize that Lmo0531 acts as a c-di-GMP receptor/effector protein similar to the I-site containing degenerate GGDEF domain proteins described earlier [48], [49]. It is peculiar that Lmo0531 is the only c-di-GMP receptor that can be predicted based on genome sequence analysis [2].

To test functions of the predicted *L. monocytogenes* DGC and PDE proteins and a single identifiable c-di-GMP receptor, we cloned and expressed these genes in *E. coli* indicator strains that respond to changes in intracellular c-di-GMP concentrations in a predictable fashion, and, where necessary, purified proteins to test their activities in vitro.

### *L. monocytogenes* PdeB-D proteins possess c-di-GMP PDE activities

**Figure 2:**
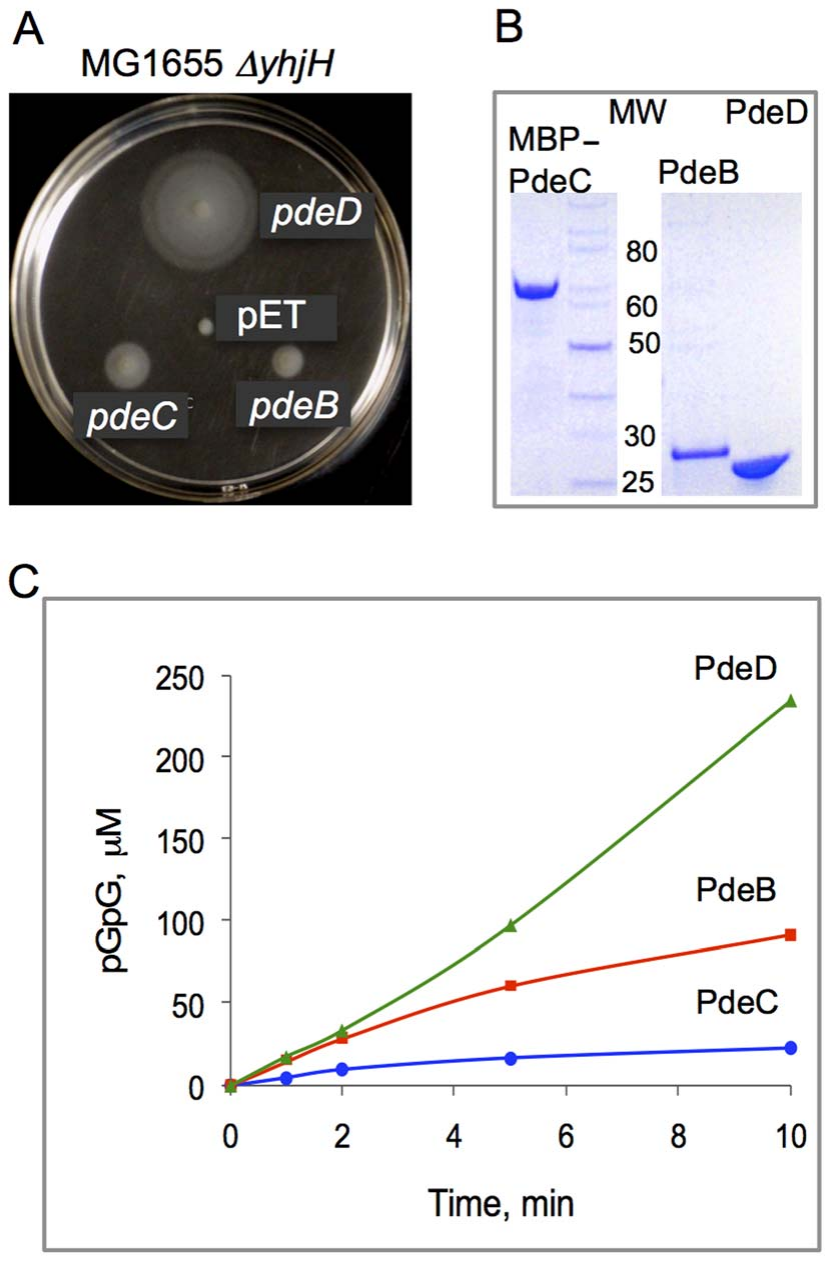
PDE activities of the *L. monocytogenes* proteins PdeB-D. **A**: Restoration of motility in semi-solid (0.25%) agar of strain MG1655 Δ*yhjH* by *L. monocytogenes* PdeB, PdeC and PdeD is indicative of their c-di-GMP PDE activities. PdeB-D were expressed as C-terminal His_6_-fusions downstream of the T7 promoter from vector pET23a. Although MG1655 does not encode a T7 RNA polymerase gene, the *pde* genes were expressed from a fortuitous promoter at sufficiently high levels to partially restore the swimming defect of MG1655 Δ*yhjH* in semi-solid agar. pET, empty vector (pET23a). **B**: Affinity purified *L. monocytogenes* PdeD (PdeD::His_6_), PdeB (PdeB::His_6_) and PdeC (MBP::PdeC) proteins used in the PDE assays. MW, molecular weight, kD. **C**: PDE activities of PdeD::His_6_, PdeB::His_6_ and MBP::PdeC monitored by the rates of formation of pGpG, the product of c-di-GMP hydrolysis. Nucleotides were measured by HPLC as described earlier [38].

We expressed *L. monocytogenes pdeB*, *pdeC* and *pdeD* in *E. coli* MG1655 Δ*yhjH*. This mutant lacks a major c-di-GMP PDE, YhjH [50], [51], and as a result, is impaired in motility in semi-solid agar [52]. We found that expression of any one of the *pde* genes was sufficient to partially restore swim zones of MG1655 Δ*yhjH* in semi-solid agar (Fig. 2A). These results are consistent with the possibility that all three proteins, PdeB, PdeC, and PdeD, function as c-di-GMP PDEs. However, overexpressed but enzymatically inactive EAL domain proteins that retain the ability to bind (but not to hydrolyze) c-di-GMP also can lower intracellular c-di-GMP concentration thus mimicking the phenotypes of overexpressed PDEs [53].

To resolve the ambiguity regarding the enzymatic activity of the PdeB-D proteins, we purified each protein and tested its ability to hydrolyze c-di-GMP in vitro. The PdeB and PdeD proteins were overexpressed and purified as N-terminal His_6_-tagged fusions. Since the His_6_-tagged PdeC fusion proved to be insoluble, PdeC was purified as a fusion to maltose-binding protein (MBP) (Fig. 2B). The ability of purified PdeB, PdeC, or PdeD to hydrolyze c-di-GMP was assessed by measuring the substrate and products of reactions over time using HPLC, as described previously [38]. Fig. 2C shows that all three recombinant proteins possess c-di-GMP PDE activities in vitro.

### *L. monocytogenes* DgcA-C proteins possess DGC activities

**Figure 3:**
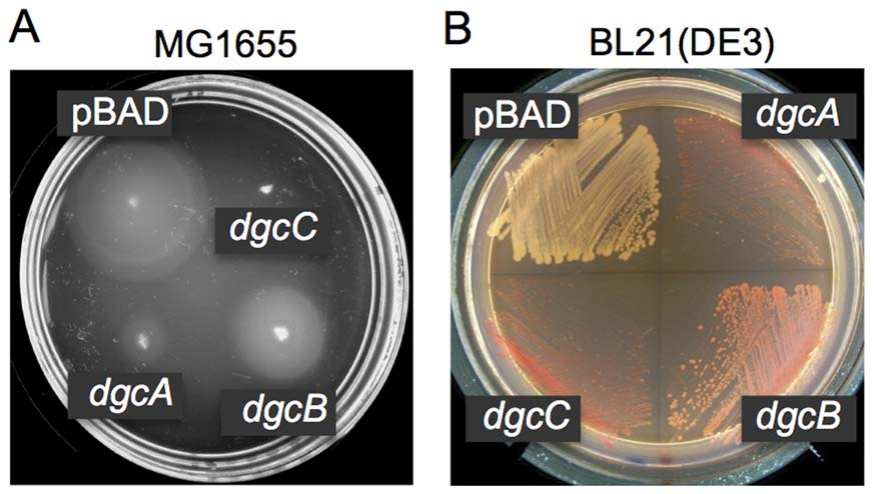
DGC activities of the *L. monocytogenes* proteins DgcA-C. **A**: Inhibition of motility in semi-solid (0.25%) agar of strain MG1655 by *L. monocytogenes* DgcA (plasmid pBAD-*dgcA*), DgcB (pBAD-*dgcB*) and DgcC (pBAD-*dgcC*) is indicative of their DGC activities. DgcA-C were expressed from the vector pBAD/Myc-His-C (pBAD). LB agar contained 0.1% arabinose. **B**: Congo red staining of the fimbriae producing strain BL21(DE3) caused by *L. monocytogenes* DgcA, DgcB and DgcC is indicative of their DGC activities. LB agar contained 0.001% arabinose.

The functionality of putative *L. monocytogenes* DGC proteins was assessed by monitoring swim zone sizes in semi-solid agar. The three *dgc* genes were cloned into the pBAD/Myc-His vector under the control of an arabinose-inducible promoter. Each of the three *dgc* genes decreased, to various degrees, the sizes of the swim zones of strain MG1655, which is highly motile in the absence of heterologous DGCs (Fig. 3A).

To exclude the possibility of nonspecific motility inhibition (e.g., due to protein toxicity), we assessed a second c-di-GMP-dependent phenotype that is independent of motility inhibition. In *E. coli* BL21 (DE3), c-di-GMP induces synthesis of curli fimbriae that can be detected by staining with Congo red dye [47]. As shown in Fig. 3B, BL21 (DE3) strains expressing each of the three Dgc proteins individually exhibited more intensely colored colonies on Congo red agar compared to the negative control expressing an empty vector. Together, these results support the prediction that the DgcA-C proteins possess DGC activity.

### *L. monocytogenes* phenotypes associated with perturbed intracellular c-di-GMP levels

Having established that *L. monocytogenes* EGD-e possesses functional components for c-di-GMP-mediated signaling, we examined phenotypes associated with elevated and decreased intracellular c-di-GMP levels. To perturb c-di-GMP levels, we expressed in the EGD-e strain two c-di-GMP metabolizing enzymes characterized by us previously, i.e., DGC (Slr1143 from *Synechocystis* sp. [37]) and PDE (YhjH from *E. coli* [51]), and assessed their role in swimming motility and EPS production. The use of heterologous proteins allowed us assess the effects of changing intracellular c-di-GMP levels without undesired changes in protein-protein interactions that may have been occured if we were to overexpress listerial DGC and PDE enzymes.

**Figure 4:**
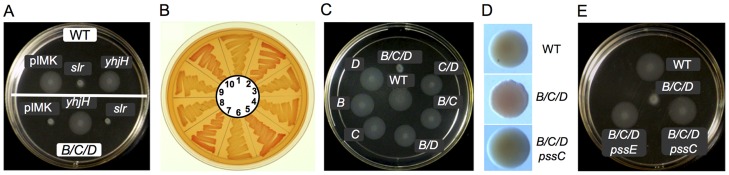
Inhibition of motility and activation of EPS production in *L. monocytogenes* by elevated levels of c-di-GMP. **A**: *Top*, Inhibition of swimming of the wild-type *L. monocytogenes* in semi-solid agar by a heterologous DGC, Slr1143. *Bottom*, Restoration of swimming in semi-solid agar of the *L. monocytogenes* **Δ** *pdeB/C/D* mutant by a heterologous PDE, YhjH. WT, wild type, EGD-e; A/B/C, **Δ** *pdeB/C/D* mutant; pIMK, WT::pIMK2 (vector control); *slr*, WT::(pIMK2::*slr1143*); *yhjH*, WT::(pIMK2::*yhjH*). **B**: Congo red staining of EPS in *L. monocytogenes*. 1, WT, wild type; 2, Δ*pdeB/C/D*; 3, Δ*pdeB/C/D* Δ*pssE*; 4, Δ*pdeB/C/D* Δ*pssC*; 5, Δ*pdeB/C/D*::pIMK2; 6, Δ*pdeB/C/D*::pIMK2::*yhjH*; 7, Δ*pdeB/C/D*::(pIMK2::*slr1143*); 8, WT::pIMK2; 9, WT::(pIMK2::*yhjH*); 10, WT::(pIMK2::*slr1143*). **C**: Deletion of all three c-di-GMP PDEs drastically inhibits motility of *L. monocytogenes* in semi-solid agar. WT, wild type strain, *B*, **Δ** *pdeB*; *C*, **Δ** *pdeC*; *D*, **Δ** *pdeD; B/D*, **Δ** *pdeB* **Δ** *pdeD*; *C/D*, **Δ** *pdeC* **Δ** *pdeD*; *B/C*, **Δ** *pdeB* **Δ** *pdeC*; *B/C/D*, **Δ** *pdeB/C/D*. **D**: Rough colony morphology and increased Congo red staining of the *L. monocytogenes* **Δ** *pdeB/C/D* mutant and rescue of the wild-type colony morphology by the **Δ** *pssC* mutation (Δ*pdeB/C/D* **Δ** *pssC*). **E**: Restoration of motility of the **Δ** *pdeB/C/D* mutant by the **Δ** *pssC* or **Δ** *pssE* mutations.

*L. monocytogenes* uses flagella for motility [54]. Expression of Slr1143 blocked swimming of strain EGD-e in semi-solid agar, whereas expression of YhjH had no effect (Fig. 4A top). Expression of Slr1143 also resulted in more pigmented *L. monocytogenes* colonies on Congo red agar, whereas expression of YhjH had no observable phenotype (Fig. 4B, sectors 10 versus 1 and 9). Later in this work we show that YhjH is expressed and functional as a PDE in *L. monocytogenes*. Therefore, we interpreted the lack of a phenotype associated with YhjH overexpression as an indication that intracellular c-di-GMP levels in strain EGD-e are already low, and that c-di-GMP does not play a significant role under the conditions used in these assays. Since *L. monocytogenes* is not known to synthesize pili, and the genome of strain EGD-e has no candidate pili genes, we hypothesized that Congo red staining was indicative of EPS production. An EPS has been suspected in some naturally occurring *L. monocytogenes* isolates [28]. Further, Tiensuu and colleagues have recently observed Congo red staining rings within *L. monocytogenes* colonies exposed to dark-light cycles [55], however the nature of the Congo red-binding extracellular polymer was not investigated.

### Construction and characterization of the *L. monocytogenes dgc* and *pde* mutants

Having identified two phenotypes associated with elevated c-di-GMP levels, we proceeded to inactivate, individually and in combination, the *L. monocytogenes pdeB-D* genes. Based on the inhibition of swim zones in semi-solid agar by the heterologous DGC, Slr1143, we expected *pdeB-D* mutations to result in smaller swim zones. However, inactivation of individual *pde* genes did not significantly affect swim zone sizes (Fig. 4C). Inactivation of pairs of *pde* genes produced relatively minor decreases in swim zones sizes, while simultaneous deletion of all three *pde* genes, *ΔpdeB/C/D*, produced a mutant severely impaired in swimming in semi-solid agar (Fig. 4C). This phenotype is similar to the phenotype of the wild type EGD-e expressing the heterologous DGC, Slr1143 (Fig. 4A top). These results suggest that the PDEs have at least partially overlapping functions in degrading intracellular c-di-GMP.

As expected, expression of Slr1143 in the triple *ΔpdeB/C/D* mutant did not affect the already inhibited motility any further (Fig. 4A bottom). However, expression of YhjH in this mutant fully restored the swim zone to the size of the wild-type strain, thus showing that YhjH is expressed and functional in *L. monocytogenes* (Fig. 4A bottom), and that motility inhibition in semi-solid agar was due to elevated c-di-GMP levels in the triple *ΔpdeB/C/D* mutant.

We proceeded to test the effects of *L. monocytogenes pde* mutations on Congo red binding. The triple *ΔpdeB/C/D* mutant showed significant accumulation of Congo red (Fig. 4B, sector 2 versus 1), similar to the wild type strain expressing Slr1143 (Fig. 4B, sector 10). Expression of YhjH, but not Slr1143, in the triple *ΔpdeB/C/D* mutant, inhibited Congo red accumulation (Fig. 4B, sector 6 versus 5 or 7). Individual *pde* mutants did not affect Congo red staining, while among double mutants, the *pdeB/C* mutant showed some staining (Figure S1).

In addition to Congo red binding, the colonies of the Δ*pdeB/C/D* mutant were found to have rough edges, compared to smooth-edged colonies of the wild type strain (Fig. 4D). The observed changes in colony morphology in the Δ*pdeB/C/D* mutant were not as pronounced as the wrinkled or rough colony morphologies reported in the proteobacterial species overexpressing EPS [56]–[59], however, when combined with enhanced Congo red binding, these changes are indicative of EPS production.

Inactivation of the *dgc* genes, individually or in combination, resulted in no observable phenotypes, just like the expression of YhjH in the wild type strain produced no phenotype. We therefore conclude that c-di-GMP plays little, if any role, in strain EGD-e grown under these laboratory conditions.

### Bioinformatics-based identification of the putative EPS biosynthesis *pssA-E* operon in *L. monocytogenes*

We searched the *L. monocytogenes* genome for EPS biosynthesis genes potentially responsible for c-di-GMP-induced Congo red binding. The *lmo0527-0531* operon, designated here *pssA-E* (polysaccharide synthesis) (Fig. 1), emerged as the prime candidate for this role based on the following reasoning. The last gene of the operon, *pssE* (*lmo0531*), encodes a degenerate GGDEF domain protein, hypothesized to function as a c-di-GMP receptor (Fig. 1). If this assumption is correct, PssE may be involved in a c-di-GMP-dependent activation of EPS synthesis, similar to activation of cellulose [1], [51], alginate [60] and Pel EPS synthesis [48] in Proteobacteria. An additional reason to implicate the *pssA-E* cluster in EPS biosynthesis was based on the presence of the putative glycosidase gene, *lmo1913*, in the *dgcA-dgcB-lmo1913-pdeC* operon that encodes enzymes for synthesis and hydrolysis of c-di-GMP (Fig. 1). Glycosidases counterbalance glycosyltransferases and are integral components of EPS synthesis and degradation apparati.

The *pssA-E* operon appears to encode enzymes associated with biosynthesis of poly-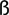-1,6-N-acetyl-D-glucosamine (PNAG) or poly-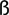-1,4-D-glucopyranose (cellulose), either of which is capable of binding Congo red, or yet another EPS. The key player in this operon is PssC (Lmo0529), which is predicted to function as type 2 glycosyltransferase responsible for the polymerization reaction. PssC shows the highest (∼30%) identity (over an ∼300 amino acid region) to the N-acetylglycosyltransferases involved in PNAG synthesis from *S. aureus* (IcaA) and *Yersinia pestis* (HmsR) [61], [62]. However, no other genes found in the staphylococcal *ica* or yersinial *hms* gene clusters are present in the *pssA-E* operon. Instead, the gene downstream of *pssC*, *pssD* (*lm0530*), encodes an ortholog of the BcsB subunit of bacterial cellulose synthases [63]. The BcsB proteins have thus far been associated exclusively with cellulose synthases, yet they are involved in the membrane passage of the polysaccharide polymer not its synthesis [64], therefore BcsB can possibly accommodate polymers of different composition than cellulose. It is noteworthy that the glycosyltransferases catalyzing cellulose synthesis also belong to type 2 glycosyltransferases, like the PNAG synthases [63]. Further, PssC shares ∼25% identity with the type 2 glycosyltransferase BcsA of the cellulose synthase complex of *Rhodobacter sphaeroides* [64]. The almost equal similarity of the listerial glycosyl transferase to PNAG- and cellulose synthases makes predictions of the composition of the listerial EPS unreliable.

### The *pssA-E* gene cluster is indeed responsible for listerial EPS synthesis

To test the involvement of the *pssA-E* gene cluster in EPS biosynthesis, we deleted the predicted glycosyltransferase gene, *pssC*, in the Δ*pdeB/C/D* background. We found that the constructed Δ*pdeB/C/D* Δ*pssC* mutant no longer bound Congo red (Fig. 4B, sector 4). This result supports the hypothesis that the *pssA-E* operon is responsible for c-di-GMP-induced EPS biosynthesis. To verify it further, we tested another bioinformatics-based prediction, i.e. that inactivation of *pssE* in the Δ*pdeB/C/D* background will also impair EPS synthesis. Indeed, the constructed Δ*pdeB/C/D* Δ*pssE* mutant did not bind Congo red either (Fig. 4B, sector 3). We conclude that PssE, a putative c-di-GMP receptor, plays a critical role in EPS synthesis. Complementation of the Δ*pdeB/C/D* Δ*pssC* and Δ*pdeB/C/D* Δ*pssE* mutants with individually cloned *pssC* and *pssE*, respectively, restored Congo red binding (data not shown) verifying that the Δ*pssC* and Δ*pssE* mutations were responsible for the mutant phenotypes. The Δ*pssC* and Δ*pssE* mutations in the Δ*pdeB/C/D* mutant background reversed the rough colony phenotype back to a smooth appearance (Fig. 4D and data not shown).

### Biochemical evidence that the PssE protein is a c-di-GMP receptor

**Figure 5:**
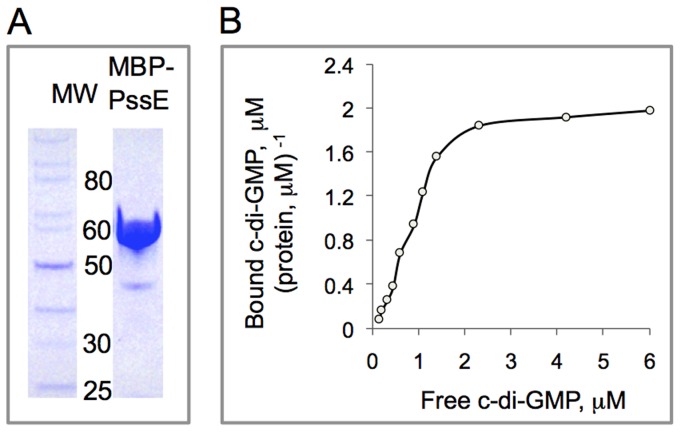
In vitro assay of c-di-GMP binding by the PssE receptor. **A**: The MBP-PssE protein purified via affinity (amylose resin) chromatography. The GGDEF domain of PssE (residues 107-285) containing the putative I-site was fused downstream of MBP, MBP::GGDEF_pssE_, and used in c-di-GMP binding assays. **B**: Saturation plot of equilibrium binding of c-di-GMP to the PssE receptor (MBP::GGDEF_pssE_). Shown is the dependence of the ratio of bound c-di-GMP per protein in the dialysis chamber, where protein alone was loaded, *versus* concentration of free c-di-GMP at equilibrium.

To test the prediction that the PssE protein acts as a c-di-GMP receptor, we overexpressed its GGDEF domain containing the I-site as an MBP fusion (MBP-GGDEF_pssE_), purified this protein (Fig. 5A) and analyzed its ability to bind c-di-GMP in vitro using equilibrium dialysis. MBP-GGDEF_pssE_ was found to bind c-di-GMP with an apparent K_d_ of 0.79±0.17 µM (Fig. 5B). This value falls within the range of physiologically relevant intracellular c-di-GMP concentrations measured in other bacteria that are believed to be in the submicromolar to low micromolar range [5]. The binding capacity of the MBP-GGDEF_pssE_ protein, B_max_, was calculated to be 2.03±0.12 µM c-di-GMP (µM protein)^−1^ indicating that each PssE molecule can bind two c-di-GMP molecules at saturation. This result is consistent with the observation of an intercalated c-di-GMP dimer bound to the I-sites of crystallized GGDEF domain proteins [2], [3]. Therefore, PssE is a *bona fide* c-di-GMP receptor that is predicted to transfer the c-di-GMP signal to activate synthesis of the listerial Pss EPS.

### C-di-GMP-induced listerial EPS promotes cell aggregation but plays limited role in biofilm formation on abiotic surfaces

**Figure 6:**
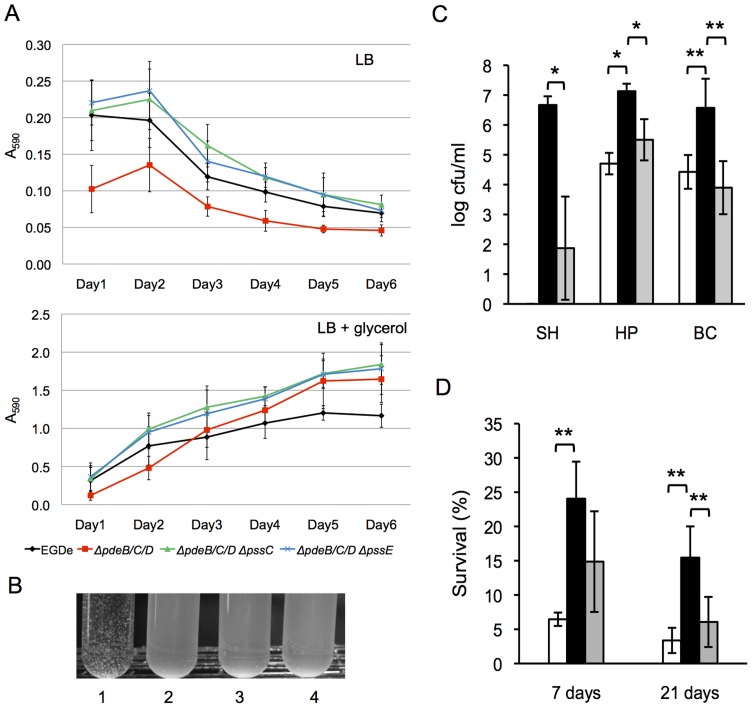
Role of the c-di-GMP-induced EPS in biofilm formation, cell aggregation, and tolerance of *L. monocytogenes* to disinfectants and desiccation. **A**: Biofilm formation of *L. monocytogenes* in 96-well polystyrene plates (measured using a Crystal violet dye-binding assay). Cultures were grown for 6 days at 30°C in LB (top panel) or LB supplemented with 3% glycerol (bottom panel). Shown are average results from two biological replicates, where each strain was grown in six wells in a replicate (i.e., six technical replicates). Black circle, wild type; red square, **Δ** *pdeB/C/D*; green triangle, **Δ** *pdeB/C/D* **Δ** *pssC*; blue cross, **Δ** *pdeB/C/D* **Δ** *pssE*. **B**: EPS-dependent *L. monocytogenes* cell aggregation (clumping) in HTM medium. Overnight cultures grown in BHI were inoculated into HTM liquid medium at A_600_ of 0.01 and incubated at 30°C with gentle shaking (rotary shaker, 125 rpm) for 48 h. 1, *ΔpdeB/C/D*; 2, wild type; 3, *ΔpdeB/C/D ΔpssC*; 4, *ΔpdeB/C/D ΔpssE*; **C**: Protective role of the c-di-GMP-inducible EPS in disinfection. Aliquots of the HTM-grown cultures were mixed with disinfectant solutions for 10 min at room temperature. Disinfection was stopped by adding a D/E neutralizing broth (Difco); the cultures were vortexed vigorously (5 min) with glass beads to break clumps and plated on BHI agar. Colonies were enumerated after a 48-h growth at 37°C. SH, sodium hydrochloride (1600 ppm); HP, hydrogen peroxide (200 mM); BC, benzalkonium chloride (100 ppm). White background, EGD-e; black, *ΔpdeB/C/D*; grey, *ΔpdeB/C/D ΔpssC*. SH, sodium hypochlorite; HP, hydrogen peroxide; BC, benzalkonium chloride. The absence of the bar for the EGD-e strain treated with SH indicates the lack of survivors. **D**: Protective role of the c-di-GMP-inducible EPS in desiccation. Aliquots of overnight cultures grown in HTM at 37°C were spun down, the supernatants were removed, and cell pellets were stored in desiccators at room temperature for the indicated periods. The pellets were rehydrated, vortexed with glass beads for better suspension and plated on BHI agar. The numbers of surviving colonies after incubation at 37°C for 24 h are plotted. In panels C and D, bars denote mean values for data from three biological replicates. *, significantly different (p<0.002), **, significantly different (p<0.02), according to Tukey test (Minitab 16 statistical software; http://www.minitab.com/).

PNAG and cellulose increase biofilm formation by the proteobacterial species on abiotic surfaces. To test the effect of c-di-GMP-induced EPS in *L. monocytogenes*, we performed a conventional Crystal violet dye-binding assay that measures the biomass of cells attached to the wells of microtiter plates following removal of liquid cultures [65]. Surprisingly, we did not observe an increase in biofilm levels in the Δ*pdeB/C/D* mutant, compared to the wild type, when these strains were grown in LB medium (where biofilm formation of strain EGD-e is low). We observed only a marginal increase in surface-attached biofilm levels in LB supplemented with glycerol (where biofilms are greatly stimulated) (Fig. 6A). Interestingly, this increase in biofilm levels was observed in all Δ*pdeB/C/D* strains grown in LB plus glycerol, whether or not they produced EPS (Fig. 6A). These results suggest that, instead of the anticipated stimulation of biofilms, listerial EPS may actually inhibit biofilm formation, at least under certain conditions. They also implicate a c-di-GMP-activated non-EPS component in biofilm stimulation. Similar to the results on polystyrene surfaces, the Δ*pdeB/C/D* mutant produced no more biofilm in LB medium on glass or metal (aluminum foil or steel coupons) surfaces than did the wild type (data not shown).

We noticed that incubation of the Δ*pdeB/C/D* mutant (but not the wild type, Δ*pdeB/C/D* Δ*pssC* or Δ*pdeB/C/D* Δ*pssE* mutants) in liquid glucose-rich minimal HTM medium resulted in cell clumping (Fig. 6B). This suggests that listerial EPS strengthens intercellular interactions but not bacterial interactions with abiotic surfaces. The *pssC* and *pssE* gene deletions in the Δ*pdeB/C/D* background completely abolished clumping, just like they decreased Congo red binding in BHI plates. This result confirms that listerial EPS is responsible for clumping.

### C-di-GMP-dependent EPS impairs *L. monocytogenes* motility in semi-solid agar

Since the EPS producing Δ*pdeB/C/D* mutant was impaired in swimming in semi-solid agar (Fig. 4C), we set out to explore the effect of EPS on motility. Surprisingly, inactivation of EPS synthesis by the *pssC* or *pssE* mutations, restored swimming of the Δ*pdeB/C/D* mutant in semi-solid agar to the wild-type levels (Fig. 4E). It therefore appears that swimming in semi-solid agar was impaired exclusively due to listerial EPS.

To gain additional insight into this issue, we analyzed the motility of the wild type, the Δ*pdeB/C/D* mutant and the Δ*pdeB/C/D* Δ*pssC* mutant in liquid medium where clumping is minimal and not detectable by the naked eye. Phase contrast microscopic observations revealed that single cells of the Δ*pdeB/C/D* and Δ*pdeB/C/D* Δ*pssC* mutants were as motile as the wild-type cells. These results favor the scenario whereby EPS accumulated on cell surfaces results in cell aggregation, which inhibits spreading of the cells in semi-solid agar.

### C-di-GMP-induced EPS significantly enhances *L. monocytogenes* tolerance to disinfectants and desiccation

EPS is known to protect bacteria from environmental insults (27). Here we evaluated the role of *L. monocytogenes* EPS in providing tolerance to disinfection and desiccation. The wild-type strain EGD-e as well as its Δ*pdeB/C/D* mutant synthesizing EPS and grown under clump-forming conditions were subjected to selected disinfectants commonly used in the food-processing industry and produce storage facilities: sodium hypochlorite (bleach), benzalkonium chloride (a quaternary ammonium compound) and hydrogen peroxide [66]. To distinguish between the contribution of EPS versus EPS-independent c-di-GMP-responsive agents, we included in the tests the Δ*pdeB/C/D* Δ*pssC* mutant characterized by elevated intracellular c-di-GMP levels but defective in EPS production.

The sodium hypochlorite treatment applied here was highly effective in killing the wild-type strain, but not the EPS producing Δ*pdeB/C/D* mutant, whose survival was approximately >10^6^-fold higher than the survival of the wild type (Fig. 6C). Tolerance of the Δ*pdeB/C/D* mutant to hydrogen peroxide and benzalkonium chloride treatments was also highly, approximately 10^2^-fold, higher, compared to the wild type or the EPS-deficient Δ*pdeB/C/D* Δ*pssC* mutant (Fig. 6C). These observations suggest that the c-di-GMP-induced EPS is a critical factor responsible for increased tolerance to these agents.

EPS also enhanced survival of *L. monocytogenes* to long-term desiccation. In this experiment, the liquid-grown cultures were centrifuged, and the pellets were kept in a desiccator for 7 or 21 days. We found that the desiccation survival rates of the EPS producing Δ*pdeB/C/D* strain were significantly higher, compared to those of the wild type or the Δ*pdeB/C/D ΔpssC* mutant (Fig. 6D). These results suggest that EPS provides superior protection not only against various commonly used disinfectants in food processing plants but also to desiccation, which may enhance listerial survival during food transportation and storage.

### Elevated c-di-GMP levels inhibit *L. monocytogenes* invasion into mammalian cells

**Figure 7:**
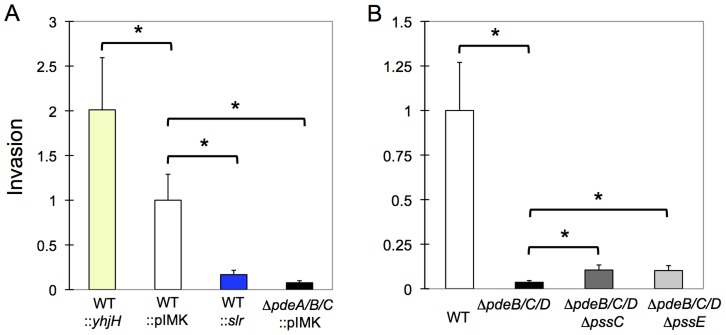
Impaired invasion of *L. monocytogenes* in HT-29 human colon adenocarcinoma cells by elevated c-di-GMP levels. **A**: Expression of the heterologous DGC, Slr1143 (WT::slr; blue bar), or deletion of the native PDEs (**Δ** *pdeB/C/D*; black), strongly inhibit listerial invasion, compared to EGD-e containing an empty vector (WT::pIMK; white), while overexpression of the heterologous PDE, YhjH (WT::yhjH; yellow), improves invasion. **B**: High intracellular c-di-GMP levels inhibit invasion more significantly than the presence of EPS. Strains shown are WT (white bar); **Δ** *pdeB/C/D* mutant (black); **Δ** *pdeB/C/D* **Δ** *pssC* (dark-grey) and **Δ** *pdeB/C/D* **Δ** *pssE* (light-grey). Plotted are values of relative invasion, compared to those of WT::pIMK (panel A) or WT (panel B). Average results from three independent tests, each performed in three replicates are shown. *, significantly different (p<0.001). Prism 5 for Mac (GraphPad) was used to perform unpaired Student's *t*-tests.

As a foodborne pathogen, *L. monocytogenes* is expected to use gut epithelial cells for primary invasion [17]–[20]. We examined the consequences of elevated c-di-GMP levels on bacterial invasion into HT-29 human colon adenocarcinoma cells. As shown in Fig. 7A, the strains with elevated c-di-GMP levels were significantly impaired in invasion, whether elevated c-di-GMP was caused by expression of the heterologous DGC, Slr1143, or by the Δ*pdeB/C/D* mutations. Consistent with the inhibitory role of c-di-GMP, invasion was increased, by approximately 2-fold, in the *L. monocytogenes* strain expressing a c-di-GMP PDE, YhjH.

Next, we tested what role the c-di-GMP-induced EPS may have played in invasion inhibition. We observed that the Δ*pdeB/C/D* Δ*pssC* and Δ*pdeB/C/D* Δ*pssE* mutants showed approximately 2–2.5-fold greater invasiveness compared to the Δ*pdeB/C/D* mutant (Fig. 7B), but remained approximately 10-fold less invasive than the wild type strain. These results suggest that while EPS moderately inhibits invasion, the major reason for the defective invasion is a c-di-GMP-induced component(s) different from EPS. The nature of this component(s) and the mechanisms through which it inhibits listerial invasion remain to be investigated.

### Elevated c-di-GMP levels reduce systemic spread of *L. monocytogenes* in mice infected via an oral route

**Figure 8:**
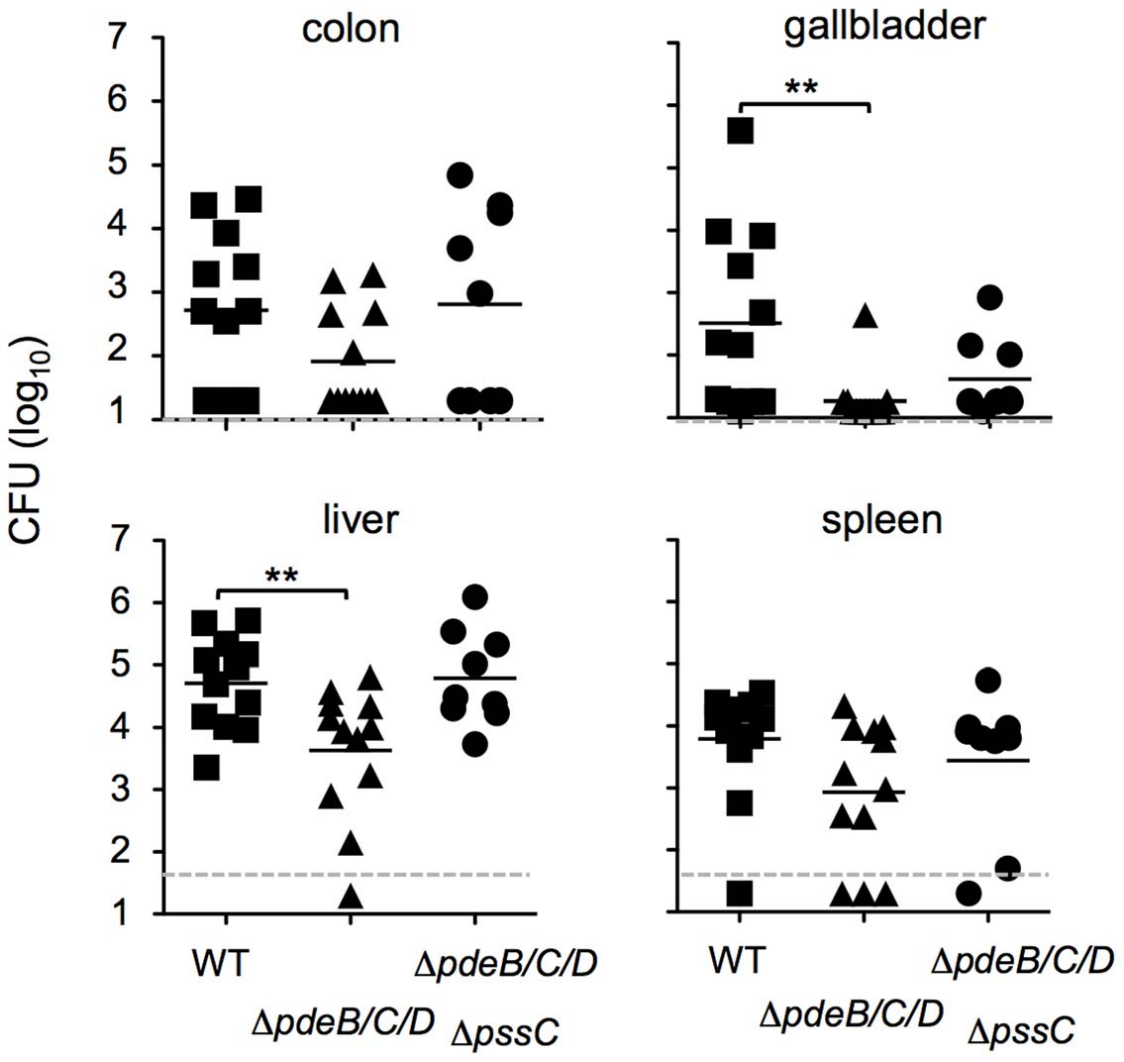
Impaired spreading of the *L. monocytogenes* Δ*pdeB/C/D* mutant to the liver and gallbladder in a foodborne model of infection. Groups of BALB/c/By/J mice were fed 5.9–7.5×10^8^ CFU of the indicated *L. monocytogenes* strains and bacterial loads were assessed 60 h post-infection. Dashed lines indicate the limit of detection for each tissue. Bars denote mean values for pooled data from three separate experiments. **, significantly different (p<0.05). Prism 5 for Mac (GraphPad) was used to perform unpaired Student's *t*-tests.

To assess the role of c-di-GMP signaling in vivo, we used a newly developed mouse model of foodborne listeriosis [67]. Groups of BALB/c/By/J mice were fed either wild-type EGD-e or the Δ*pdeB/C/D* mutant, and the bacterial load in various tissues was assessed 60 h post infection. There was no significant difference in colonization of the ileum, colon or spleen at this time point (Fig. 8). However, the Δ*pdeB/C/D* triple mutant was significantly impaired in colonizing both the liver and the gallbladder. The decreased bacterial load in the liver appears to be linked to EPS, since the Δ*pssC* mutation in the Δ*pdeB/C/D* background restored the bacterial load to the wild-type level (Fig. 8). In fact, no significant differences in bacterial loads in the liver were observed when the same *L. monocytogenes* strains were injected intravenously (Fig. S2), suggesting that increased levels of c-di-GMP may alter the ability of the bacteria to disseminate from the gut.

## Discussion

We predicted that the *L. monocytogenes* EGD-e genome encodes three GGDEF domain DGCs, one inactive GGDEF domain protein and three EAL domain PDEs, and verified this prediction by a combination of genetic and biochemical tests. Interestingly, all of the enzymes involved in c-di-GMP metabolism are highly conserved not only in the genomes of *L. monocytogenes* isolates but also in other *Listeria* species, e.g. *L. innocua*, *L. ivanovii*, *L. seeligeri*, and *L. welshimeri*. The high conservation of these proteins implies that c-di-GMP signaling pathways play important roles in the evolutionary success of *Listeria*. Such conservation is striking in light of the flexibility in the organization of c-di-GMP signaling pathways observed in other Firmicutes. For example, in the genus *Bacillus*, the number of enzymes involved in c-di-GMP synthesis and hydrolysis varies from three to eleven; it varies from eight to forty in the genus *Clostridium* (http://www.ncbi.nlm.nih.gov/Complete_Genomes/SignalCensus.html).

We discovered that c-di-GMP regulation affects at least two targets in *L. monocytogenes*. One of these targets is a novel EPS (Fig. 4B, 4D, 6B). This finding resolves the long-standing controversy regarding the ability of listeria to produce EPS [27], [28]. The second (and possibly additional) target of c-di-GMP regulation, whose identity remains unknown, appears to be responsible for the drastic inhibition of listerial invasiveness in mammalian cells (Fig. 7), modest stimulation of biofilm formation on abiotic surfaces in LB supplemented with glycerol (Fig. 6A) and lower pathogen accumulation in certain mouse organs following oral infection (Fig. 8).

Here, we revealed that the c-di-GMP induced EPS is synthesized by the *pssA-E* operon. The composition of the listerial EPS is difficult to predict because, while some Pss proteins share similarity to the components of cellulose synthases and PNAG synthases, other components are unique. Interestingly, in contrast to cellulose or PNAG, both of which promote biofilm formation on abiotic surfaces [61], [62], [64], [68], listerial EPS either does not affect or inhibits biofilm formation on abiotic surfaces. Instead, it promotes cell aggregation, in minimal media (Fig. 6B). These observations favor the hypothesis that the composition of listerial EPS is different from cellulose or PNAG.

In this study, we identified the mechanism through which c-di-GMP activates EPS synthesis in *L. monocytogenes*. C-di-GMP binds to the I-site receptor PssE, whose gene is located in the *pss* operon, and whose function is essential for EPS biosynthesis. Bacterial cellulose synthases studied thus far are activated via c-di-GMP-binding PilZ domains linked to the C-termini of BcsA subunits [1], [51], [64]. The PNAG synthase of *E. coli* is activated by c-di-GMP binding to two subunits, PgaD and PgaC, one of which, PgaD, is proteolytically degraded in the absence of c-di-GMP [69]. Perhaps the most similar c-di-GMP-dependent mechanism to that operating in *L. monocytogenes* Pss synthase involves the *Pseudomonas aeruginosa* Pel EPS synthase, which is activated via an I-site c-di-GMP receptor protein [48], [70].

We showed that the *L. monocytogenes* Pss EPS is responsible for multiple phenotypes, i.e., cell aggregation, decreased motility in semi-solid media, moderate inhibition of invasiveness in mammalian cells, and drastically elevated tolerance to disinfectants and desiccation. The latter effects of c-di-GMP-induced listerial EPS are particularly noteworthy in light of the increasing frequency of listerial outbreaks associated with produce. EPS may contribute to enhanced survival of listeria on produce surfaces during washing with disinfectants as well as during transporation and storage of listeria-contaminated produce. It is also possible that listerial EPS contributes to bacterial survival in food-processing facilities. It will be interesting to investigate whether listerial strains from the recent outbreaks synthesize EPS, and what role, if any, it may have played in their survival and disease causing abilities.

C-di-GMP-induced motility inhibition is common in Proteobacteria. One of the best-understood mechanisms of c-di-GMP-induced motility inhibition involves YcgR, the PilZ-domain c-di-GMP backstop brake that operates in *E. coli* and related enteric bacteria. YcgR binds to the flagellar switch complex and, at elevated c-di-GMP concentrations, introduces a rotational bias that decreases the frequency of flagella reversals and therefore, the frequency of changes in swimming direction [52], [71], [72]. The smooth, almost unidirectional, swimming results in bacteria being trapped in blind alleys of semi-solid agar [73]. YcgR may also slow down rotating flagella [74]. A similar mechanism has been proposed for a PilZ domain protein in *B. subtilis*, however important details have yet to be elucidated [31]. A different mechanism of c-di-GMP-induced motility inhibition was described in *Caulobacter crescentus*, where a PilZ domain receptor affects the abundance of a flagellum assembly regulatory subunit [75]. *B. subtilis* has yet another mechanism of motility inhibition that involves a bifunctional protein EpsE that acts as a glycosyl transferase involved in EPS synthesis and as a molecular clutch that disengages the flagellum rotor from the membrane-localized energy-supplying stator [76], [77]. Whether an EpsE-like clutch operates in *L. monocytogenes* remains unknown, however it is clear that no clutch or break is induced by c-di-GMP because liquid-grown cells show no obvious motility defects. The most striking observation is that inactivation of Pss synthesis is sufficient to restore motility in semi-solid agar. Therefore, listerial spreading in semi-solid agar appears to be inhibited due to cell aggregation and possibly flagella trapping in the EPS. Recently, a similar mechanism has been described in *S. enterica*, which at high c-di-GMP levels, secretes cellulose [78].

Listerial EPS inhibits bacterial invasiveness in mammalian cells, however, only modestly, 2–2.5-fold, whereas an as yet unidentified c-di-GMP pathway is responsible for a much larger component of invasiveness inhibition. The composition of this new c-di-GMP signaling pathway remains unknown. In this regard, it is noteworthy that PssE is the only c-di-GMP receptor that can be predicted based on the genome sequence analysis. *Listeria* lack other identifiable c-di-GMP receptor proteins or c-di-GMP-sensing riboswitches (reviewed in [2], [3], [6]).

In addition to uncovering the role of c-di-GMP in vitro, we tested its role in virulence using a recently developed food borne mouse disease model that closely mimics human infection (67). We found that elevated c-di-GMP levels decreased listerial infection in the liver, and that this defect could be restored by abolishing EPS biosynthesis. Thus, it is possible that c-di-GMP induced EPS impairs the ability of *L. monocytogenes* to either efficiently disseminate from the intestine or to replicate and spread from cell-to-cell in hepatocytes. While we observed a significant defect in the ability of the Δ*pdeB/C/D* mutant to invade HT-29 colon carcinoma cells in vitro, there was no difference in the ability of the Δ*pdeB/C/D* mutant to colonize the murine intestines, compared to the wild type strain. Thus, increased c-di-GMP levels may impair the direct invasion of intestinal epithelial cells mediated by InlA/E-cadherin interactions, but does not significantly impede the ability of *L. monocytogenes* to translocate across the gut mucosa barrier, presumably because the bacteria use alternate mechanisms of invasion. *L. monocytogenes* can transcytose across M cells, specialized epithelial cells that are found both overlying Peyer's patches and scattered elsewhere throughout the epithelium [79]–[81]. It is also possible that specialized subsets of dendritic cells in the intestinal lamina propria can engulf *L. monocytogenes* by extending dendrites into the gut lumen, a process that has been demonstrated during oral *S. enterica* infection [82], [83].

Another issue pertaining to this study concerns the role of cyclic dinucleotides as bacterial biomarkers recognized by the innate immune system and in the stimulation of the host intracytoplasmic surveillance response. Recently, the listerial second messenger c-di-AMP, which is structurally related to c-di-GMP, has been shown to be secreted into the cytosol of infected mammalian cells where it triggers interferon (IFN) production via the STING-signaling cascade [84]–[86]. A robust IFNβ response promotes the growth of *L. monocytogenes* administered intravenously [87]. We showed here that the Δ*pdeB/C/D* mutant, which likely has the highest c-di-GMP production achievable during *L. monocytogenes* intracellular growth, was overall less infective following oral infection. This suggests that elevated c-di-GMP levels play a negative role in *L. monocytogenes* virulence, in an apparent contrast with the role of c-di-AMP [87]. However, it is premature to draw definitive conclusions because (i) while c-di-AMP secretion enhances intracellular growth and spread of *L. monocytogenes*, overproduction of c-di-AMP suppresses listerial virulence [88], and (ii) individual c-di-GMP signaling pathways often play different, even opposite, roles in host-pathogen interactions [reviewed in ref. 2]. Therefore, more detailed analysis of c-di-GMP synthesis and secretion during listerial intracellular growth will be required to figure out the roles of c-di-GMP signaling pathways in *L. monocytogenes* virulence.

In addition to questions regarding intracellular growth and spread of listeria in different organs, our study raises numerous other questions pertaining to c-di-GMP signaling in listeria. How does c-di-GMP signaling inhibit cell invasion? What signals control c-di-GMP synthesis? Can c-di-GMP signaling pathways be manipulated to inhibit listerial cell invasion? What is the composition of the Pss EPS? Does this EPS contribute to listerial colonization of produce surfaces? Is it made by the *L. monocytogenes* strains from recent produce-associated outbreaks? Does it affect survival of such strains during disinfection in food processing facilities and desiccation during transportation and storage? These questions will have to be addressed in the future.

## Materials and Methods

### Ethics statement

This work was performed in accordance with the recommendations in the Guide for the Care and Use of Laboratory Animals published by the National Institutes of Health. All procedures were approved by the Institutional Animal Care and Use Committee (IACUC) at the University of Kentucky (permit number A-3336-01).

### Bacterial strains, plasmids and growth conditions

The bacterial strains and plasmids used in this study are listed in Table 1. The primers used in this study are listed in Table S1. *E. coli* was routinely grown in LB medium supplemented with appropriate antibiotics at 25, 30 or 37°C, as indicated. *L. monocytogenes* was grown in Brain Heart Infusion (BHI) medium (Difco), HTM (minimal medium containing 3% glucose) [89] or LB, supplemented with appropriate antibiotics at 25, 30, 37 or 42°C, as indicated.

**Table 1:** Strains and plasmids used in this study.

Strain and plasmid	Relevant genotype or description	Reference or source
Strains		
*Escherichia coli*		
DH5α	Strain used for plasmid maintenance and overexpression of MBP-fusions	Lab collection
BL21(DE3) pLysS	Strain used for overexpression of the His_6_-fusions	Invitrogen
MG1655	Wild type	ATCC 700926a*
MG1655 Δ*yhjH*	MG1655 Δ*yhjH::Km^r^*	[72]
*Listeria monocytogenes*		
EGD-e	Wild type	ATCC BAA-679
Δ*dgcAB*	In-frame deletion of the *dgcAB* genes	This study
Δ*dgcC*	In-frame deletion in *dgcC*	This study
Δ*dgcA/B/C*	Deletion of the *dgcAB* and *dgcC* genes	This study
Δ*pdeB*	In-frame deletion in *pdeB*	This study
Δ*pdeC*	In-frame deletion in *pdeC*	This study
Δ*pdeD*	In-frame deletion in *pdeD*	This study
Δ*pdeB/C*	Deletion of the *pdeB* and *pdeC* genes	This study
Δ*pdeB/D*	Deletion of the *pdeD* and *pdeB* genes	This study
Δ*pdeC/D*	Deletion of the *pdeD* and *pdeC* genes	This study
Δ*pdeB/C/D*	Deletion of the *pdeB*, *pdeC* and *pdeD* genes	This study
Δ*pdeB/C/D* Δ*pssC*	Δ*pdeB/C/D* and in-frame deletion in *pssC*	This study
Δ*pdeB/C/D* Δ*pssE*	Δ*pdeB/C/D* and in-frame deletion in *pssE*	This study
Δ*pdeB/C/D*::pIMK	Δ*pdeB/C/D*::pIMK2	This study
Δ*pdeB/C/D::yhjH*	Δ*pdeB/C/D*::(pIMK2::*yhjH*)	This study
Δ*pdeB/C/D::slr*	Δ*pdeB/C/D*::(pIMK2-*slr1143*)	This study
WT::pIMK	EGD-e::pIMK2	This study
WT::*slr*	EGDe::(pIMK2::*slr1143*)	This study
WT::*yhj*H	EGDe::(pIMK2::*yhjH*)	This study
**Plasmids**		
pAD1-cYFP	Plasmid for *L. monocytogenes* labeling	[96]
pBAD/Myc-His-C	Vector for arabinose-inducible expression	Invitrogen
pBAD-*dgcA*	pBAD::*dgcA*	This study
pBAD-*dgcB*	pBAD::*dgcB*	This study
pBAD-*dgcC*	pBAD::*dgcC*	This study
pET23a	Vector for T7-inducible His_6_-fusion protein overexpression	EMD Biosciences
pET-*pdeD*	pET23a::*pdeD*	This study
pET-*pdeB*	pET23a::*pdeB*	This study
pET-*pdeC*	pET23a::*pdeC*	This study
pIMK2	*L. monocytogenes* chromosome integrated expression vector	[97]
pIMK::*slr*	pIMK2::*slr1143*	This study
pIMK::*yhj*H	pIMK2::*yhj*H	This study
pKSV7	Vector for gene replacements in *L. monocytogenes*	[90]
pKSV7-Δ*dgcAB*	Plasmid for in-frame deletion of *dgcAB*	This study
pKSV7-Δ*dgcC*	Plasmid for in-frame deletion of *dgcC*	This study
pKSV7-Δ*pdeB*	Plasmid for in-frame deletion of *pdeB*	This study
pKSV7-Δ*pdeC*	Plasmid for in-frame deletion of *pdeC*	This study
pKSV7-Δ*pdeD*	Plasmid for in-frame deletion of *pdeD*	This study
pKSV7-Δ*pssC*	Plasmid for in-frame deletion of *pssC*	This study
pKSV7-Δ*pssE*	Plasmid for in-frame deletion of *pssE*	This study
pLysS	Lysozyme expressing plasmid for T7-expression systems	Invitrogen
pMAL-c2x	Vector for MBP-fusion protein overexpression	NEB
pMAL-*pdeC*	pMAL-c2x::*pdeC*	This study
pMAL-GGDEF_pssE_	pMAL-c2x::*pssE*(GGDEF domain)	This study

*ATCC, American Type Culture Collection.

### Plasmid and mutant construction

Genomic DNA of *L. monocytogenes* EGD-e was purified from bacterial cells using a Bactozol kit (Molecular Research Center, OH). *L. monocytogenes* genes were PCR amplified using genomic DNA, *Vent* DNA polymerase (New England Biolabs), and gene-specific primers (Table S1). PCR fragments were gel purified with the Gel Purification kit (Qiagen), digested with the appropriate restriction enzymes, and cloned into vector pMAL-c2x (New England Biolabs) in strain DH5α or into vector pET23a (Invitrogen) in strain BL21(DE3) containing pLysS (Invitrogen).

In-frame deletions in the *pdeB/C/D*, *dgcA/B/C*, *pssC* and *pssE* genes were generated by site-directed mutagenesis by splice-by-overlap extension PCR. The PCR products containing genes with in-frame deletions were cloned into the temperature-sensitive shuttle vector pKSV7 [90]. The recombinant sequences were used to replace the corresponding wild type sequences in the chromosome of the *L. monocytogenes* EGD-e strain by allelic exchange, as previously described [90]. *L. monocytogenes* was electroporated as described earlier [91].

### Motility and Congo red dye binding assays

The analysis of swimming in semi-solid agar was performed essentially as described [51]. Briefly, 2 µl of overnight cultures was inoculated onto soft agar plates containing 0.25% agar, 1% tryptone and 0.5% NaCl. Diameters of the swimming zones were assessed after 6-h incubation at 37°C for *E. coli* and 12–18-h incubation at 30°C for *L. monocytogenes*.

For Congo red binding assays, LB (*E. coli*) or BHI (*L. monocytogenes*) agar plates containing 40–80 µg ml^−1^ Congo red were incubated at 30°C for 48–72 h.

### Biofilm assays

Surface-adhered biofilm formation was assayed in a 96-well format using a modified version of a previously published protocol [92]. Briefly, overnight cultures grown in BHI at 30°C (A_600_, 2.5–3.5) were diluted into freshly made BHI, LB or LB supplemented with 3% glycerol to an initial A_600_ of 0.05–0.1, and 150 µl aliquots of each culture were inoculated into each of six wells. Biofilms attached to wells were measured following growth for 1–6 days at 30°C. Biofilms were stained with a 0.1% aqueous solution of Crystal violet dye, which was subsequently dissolved in 33% acetic acid and quantified by measurement of A_595_ [65].

### Protein overexpression and purification

For purification of PdeB::His_6_ and PdeD::His_6_, isopropyl-β-D-thiogalactopyranoside (IPTG) (final concentration, 0.2 mM) was added to exponentially (A_600_, 0.6–0.7) growing cultures of *E. coli* BL21 (DE3) pLysS containing appropriate overexpression plasmids. After 2 to 4 h of induction at 30°C, the cells were chilled to 4°C and collected by centrifugation. The cell pellets were resuspended in buffer (pH 8.0) containing 300 mM NaCl, 50 mM NaH_2_PO_4_, and 10 mM imidazole and protease inhibitors (phenylmethylsulfonyl fluoride and P8849 protease inhibitor cocktail) at the concentrations specified by the manufacturer (Sigma-Aldrich). The cell suspensions were passed through a French press mini-cell (Spectronic Instruments, NJ), followed by a brief sonication using a Sonifier 250 (Branson Ultrasonics, CT). The crude cell extracts were centrifuged at 15,000× *g* for 45 min. Soluble protein fractions were collected and mixed with preequilibrated Ni^2+^ resin (Qiagen) for 1 h at 4°C, which was placed into a column and extensively washed with the resuspension buffer containing 20 mM imidazole. The proteins were subsequently eluted using 200 mM imidazole. The buffer was exchanged with PDE buffer [38] using desalting columns according to the instructions of the manufacturer (Pierce Biotechnology). Protein purity was assessed by SDS-PAGE and protein concentration was determined using a BCA protein assay kit (Pierce Biotechnology).

For purification of MBP::PdeC and MBP::GGDEF_pssE_ fusions, IPTG (final concentration, 0.2 mM) was added to exponentially (A_600_, 0.6–0.8) growing *E. coli* DH5α containing appropriate plasmids. After 2-h induction, cells were collected by centrifugation. Cell pellets were resuspended in a buffer containing 200 mM NaCl, 0.5 mM EDTA, 5 mM MgCl_2_, 20 mM Tris-HCl (pH 7.6), and 5% glycerol that also contained protease inhibitors. Following cell disruption and clearing of the crude cell extracts, as described above, soluble protein fractions were mixed with pre-equilibrated amylose resin (New England Biolabs) for 1 h at 4°C, which was subsequently extensively washed with the resuspension buffer. MBP fusions were eluted with maltose and the buffer was exchanged for PDE or c-di-GMP binding assay [51] buffer using desalting columns.

### PDE assays

Assays were performed essentially as described by Schmidt et al. [38]. Briefly, a PDE enzyme (1–5 µM) was added to PDE reaction buffer (final volume, 100 µl) containing 250 µM c-di-GMP, and the reaction was allowed to proceed at 37°C. Aliquots were withdrawn at various time points; the reaction was stopped by addition of CaCl_2_ (final concentration, 10 mM), and the sample was boiled for 3 min and centrifuged. The supernatant was then filtered through a 0.22-µm filter, and the reaction products were analyzed by reversed-phase HPLC (Summit HPLC system; Dionex) using a Supelcosil LC-18-T column (Sigma-Aldridge). The buffer system and gradient elution program were described previously [37].

### Equilibrium dialysis

Equilibrium dialysis experiments were performed as described earlier [51]. Briefly, MBP-GGDEF_PssE_ (20 µM) was injected into one of the two chambers of a Dispo-Biodialyzer cassette (10 kD cutoff, The Nest Group, MA) filled with dialysis buffer. c-di-GMP (concentrations from 1 to 50 µM) was injected into the opposite cell of the cassette. The cassettes were maintained for 25 h at room temperature under agitation, after which samples from each chamber were withdrawn, boiled for 3 min, centrifuged, and supernatants were filtered through a 0.22-µm microfilter. The nucleotide concentrations were quantified by HPLC. Binding constants were calculated by the GraphPad Prism software, version 4.03 (GraphPad Software, San Diego, CA) using a nonlinear regression model.

### Invasion assay

*L. monocytogenes* invasion properties were analyzed using a gentamicin-based assay with HT-29 human colon adenocarcinoma cell monolayers in 24-well plates, essentially as described [93], [94]. Briefly, overnight cultures of *L. monocytogenes* grown in BHI at 37°C were centrifuged, washed and resuspended in DMEM medium. Monolayers of HT-29 cells were inoculated with 100 µl of the *L. monocytogenes* suspensions (∼5×10^8^ CFU ml^−1^) at a multiplicity of infection of 100 and incubated for 1.5 h at 37°C in a 7% CO_2_ atmosphere. The monolayers were then washed and incubated in the presence of 100 µg gentamicin ml^−1^ (final concentration) for 1.5 h. Following this incubation, the cell monolayers were washed again and lysed with 0.1% Triton X-100. Appropriate dilutions were plated on BHI plates for enumeration of intracellular bacteria. Each experiment was done in triplicate, and experiments were performed at least three times independently. Statistical analysis was performed by using Tukey's test at *p* of <0.05.

### Foodborne infection of mice

Female BALB/c/By/J mice were purchased from The Jackson Laboratory (Bar Harbor, ME) at 5 weeks of age and used in experiments when they were 6–9 weeks old. Mice were maintained in a specific pathogen free facility at the University of Kentucky and all procedures were performed in accordance with IACUC guidelines. Aliquots of early stationary phase bacteria were prepared and stored at −80°C. To prepare the inoculum, aliquots were thawed on ice, cultured standing in BHI broth for 1.5 h at 30°C, washed once in PBS and then suspended in 5 µl of melted, salted butter (Kroger) and used to saturate a 2–3 mm piece of white bread (Kroger). Infection by the natural feeding route was carried out at night as described [67]. Briefly, mice were given unrestricted access to water but denied food for 22 h, then placed in an empty cage, and given 5–10 minutes to pick up the contaminated bread piece and eat all of it. Mice were then returned to their original cages with raised wire flooring to prevent coprophagy, and normal mouse chow was replenished.

### Processing of tissue samples

Colon contents were removed by squeezing with sterile forceps and then flushing with 8 ml of PBS through a 25 g needle. Washed tissues were cut longitudinally and homogenized for 1 min in 2 ml of sterile water using a PowerGen 1000 homogenizer (Fisher) at 60% power. The total number of cell-associated (adherent plus intracellular) *L. monocytogenes* cells was determined by plating serial dilutions on BHI agar supplemented with 15 g LiCl l^−1^ and 10 g glycine l^−1^ (BHI/L+G). Colonies were counted after 48 h incubation at 37°C. This selective agar inhibited the growth of most intestinal microbiota; suspect colonies were confirmed to be *L. monocytogenes* by plating on CHROMagar *Listeria* plates (Becton Dickinson). Spleens and livers were harvested aseptically and homogenized for 30 sec in 2 ml of sterile water. Gallbladders were ruptured with sterile scissors in a microfuge tube containing 1 ml of sterile water and vortexed for 30 sec. Dilutions of each tissue were plated on BHI/L+G agar.

### Disinfection and desiccation tolerance

Solutions of sodium hypochlorite, hydrogen peroxide and benzalkonium chloride (Sigma-Aldrich and Sigma Life Sciences) were prepared in sterile phosphate-buffered saline with disinfection concentrations of 1600 ppm, 200 mM, and 100 ppm, respectively. Cultures were grown in HTM with 3% glucose at 37°C for 24 h, at which point small uniform clumps are formed by the Δ*pdeB/C/D* strain. Aliquots (250 µl; 10^8^ cfu/ml) of these cultures were mixed with disinfectants at 1∶1 vol ratios in small glass tubes that also contained 0.1 g of acid washed glass beads (Sigma Life Sciences). Following a 10-min exposure to disinfectants at room temperature, D/E neutralizing broth (500 µl; Difco) was added [95]. Samples were vigorously vortexed (for 5 min) and clumps of the Δ*pdeB/C/D* strain were dispersed due to the action of the glass beads. Serial dilutions were plated on BHI agar and colonies were counted following a 48-h incubation at 37°C.

To assess desiccation tolerance, strains were grown as described above. One milliliter of cultures (5×10^8^ cfu/ml) was centrifuged in 1.5 ml eppendorf microtubes containing 0.1 g glass beads. After supernatant removal, the tubes were stored at room temperature in a desiccator jar containing anhydrous calcium sulfate. After 7 and 21 days, the pellets were resuspended in phosphate buffered saline, vigorously vortexed, and plated on BHI agar. Colonies were counted following 48-h incubation at 37°C.

## Supporting Information

Figure S1 **Congo red staining of EPS in the** ***L. monocytogenes pde*** **mutants.** Congo red staining shows partially redundant functions of PDEs. Presence of at least one PDE is sufficient to prevent full-scale induction of the EPS synthesis. 1, WT, wild type; 2, Δ*pdeB/C/D*; 3, Δ*pdeB/C*; 4, Δ*pdeC/D*; 5, Δ*pdeB/D*; 6, Δ*pdeD*; 7, Δ*pdeB*; 8, Δ*pdeC*.
(PDF)

Figure S2 **Effects of c-di-GMP on intravenous** ***L. monocytogenes*** **infections.** Cyclic di-GMP levels do not affect growth in the liver and spleen of *L. monocytogenes* delivered intravenously. **A**: Female BALB/c/By/J mice (n = 4) were co-infected intravenously with a 1∶1 mixture of wild type made chloramphenicol-resistant (Cm^R^) by chromosomal insertion of pAD1-cYFP (Table 1) and Δ*pdeB/C/D* mutant (∼600 CFU of each for a total inoculum of 1.2×10^3^ CFU). Three days post-infection, spleens and livers were harvested aseptically, homogenized, diluted and plated on BHI agar with or without the presence of 7 µg/ml of chloramphenicol. The number of chloramphenicol-sensitive (Cm^S^) Δ*pdeB/C/D* CFU was determined by subtracting the number of (Cm^R^) colonies from the total CFU found on plates without antibiotic. Competitive index (CI) ratios were determined by dividing the number of Cm^S^ *ΔpdeB/C/D* CFU by the number of Cm^R^ wild type CFU recovered from each tissue. **B**: A competition experiment performed with the Cm^R^ wild type and the strain expressing the *E. coli* PDE, YhjH. WT, chloramphenicol-resistant (Cm^R^) derivative of strain EGD-e; pIMK::*yhjH*, EGD-e with integrated plasmid pIMK2 expressing *E. coli* PDE, YhjH (Table 1).
(PDF)

Table S1 **Primers used in this study.**
(DOC)
