# Supplementary figures and images for: Cyclic di-GMP-dependent Signaling Pathways in the Pathogenic Firmicute Listeria monocytogenes
Source: PLoS Pathog. 2014 Aug 7;10(8):e1004301. doi: 10.1371/journal.ppat.1004301 (PMC4125290; doi:10.1371/journal.ppat.1004301)

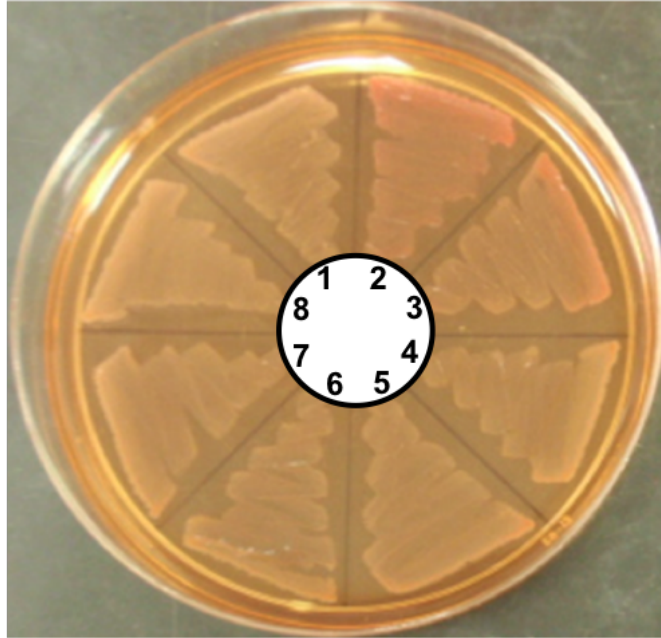

Supplement: Figure S1 — Congo red staining of EPS in the L. monocytogenes pde mutants. Congo red staining shows partially redundant functions of PDEs. Presence of at least one PDE is sufficient to prevent full-scale induction of the EPS synthesis. 1, WT, wild type; 2, ΔpdeB/C/D; 3, ΔpdeB/C; 4, ΔpdeC/D; 5, ΔpdeB/D; 6, ΔpdeD; 7, ΔpdeB; 8, ΔpdeC. (PDF) [file ppat.1004301.s001.pdf]

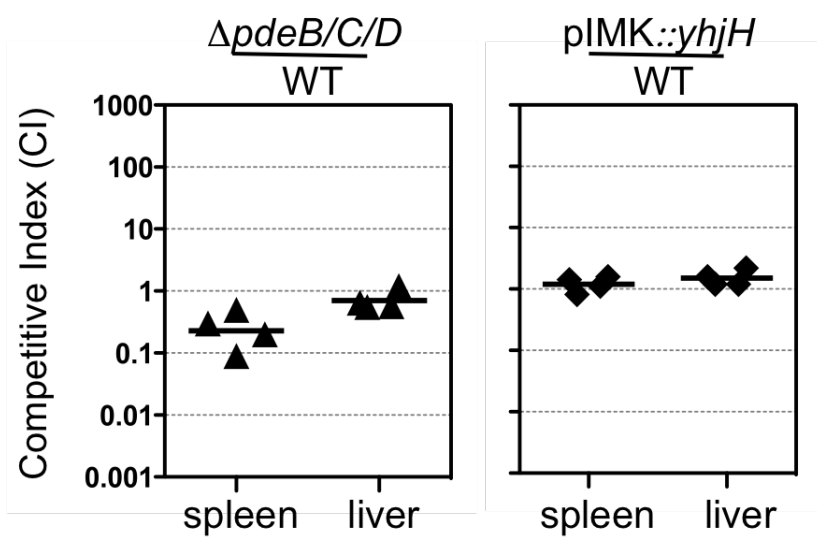

Supplement: Figure S2 — Effects of c-di-GMP on intravenous L. monocytogenes infections. Cyclic di-GMP levels do not affect growth in the liver and spleen of L. monocytogenes delivered intravenously. A: Female BALB/c/By/J mice (n = 4) were co-infected intravenously with a 1∶1 mixture of wild type made chloramphenicol-resistant (CmR) by chromosomal insertion of pAD1-cYFP (Table 1) and ΔpdeB/C/D mutant (∼600 CFU of each for a total inoculum of 1.2×10^3^ CFU). Three days post-infection, spleens and livers were harvested aseptically, homogenized, diluted and plated on BHI agar with or without the presence of 7 µg/ml of chloramphenicol. The number of chloramphenicol-sensitive (CmS) ΔpdeB/C/D CFU was determined by subtracting the number of (CmR) colonies from the total CFU found on plates without antibiotic. Competitive index (CI) ratios were determined by dividing the number of CmS ΔpdeB/C/D CFU by the number of CmR wild type CFU recovered from each tissue. B: A competition experiment performed with the CmR wild type and the strain expressing the E. coli PDE, YhjH. WT, chloramphenicol-resistant (CmR) derivative of strain EGD-e; pIMK::yhjH, EGD-e with integrated plasmid pIMK2 expressing E. coli PDE, YhjH (Table 1). (PDF) [file ppat.1004301.s002.pdf]
