# Supplementary material for: Cyclic di-GMP-dependent Signaling Pathways in the Pathogenic Firmicute Listeria monocytogenes
Source: PLoS Pathog. 2014 Aug 7;10(8):e1004301. doi: 10.1371/journal.ppat.1004301 (PMC4125290; doi:10.1371/journal.ppat.1004301)
Supplement: Table S1 — Primers used in this study. (DOC) [file ppat.1004301.s003.doc]

**Table S1.**

In-frame deletions

dgcA.A gatcacctgcagccgtcctgtatctccttcgagtg

dgcA.B gcgaatatgtacttgcgccatgctgttccataatgaatcaag

dgcA.C tggaacagcatggcgcaagtacatattcgcgaaacagaaccag

dgcA.D tcgatagaattcgcagttacaaagaacagcaagaaatactcg

dgcB.A gatcacctgcagcctttgatgctgcattcaaccatg

dgcB.B ctgatgaacagcaatattttggaaccaattagggcgatattgtc

dgcB.C aattggttccaaaatattgctgttcatcagggggaac

dgcB.D tcgatagaattctctgttgcattgcttggatatttagatagc

dgcC.A atagatctgcagcggcatctggaatggggcaacaaattgc

dgcC.B taacgttctagacaactgttcgaccaaaaagcatc

dgcC.C attgcatctagagtatgtattgcagacggaaattagtctg

dgcC.D gtcataggtacccttacctcgccagtttcaagcactcg

pdeB.A gatcacctgcagtgaacacggatagcattgtcag

pdeB.B ggctcatacaatggagctcgagatatccatcttatccctc

pdeB.C atggatatctcgagctccattgtatgagccggataaatag

pdeB.D tcgatagaattccgactaccatatgacgctagc

pdeC.A gatcacaagctttcagggggaactcgaatgaaacg

pdeC.B cactgactgcagggagggctttttcatttgtccaac

pdeC.C gtacacctgcagtaagaattaaaaagaaaatctgcttattgtcag

pdeC.D tcgataggatcccatcaattgcgcactaagttgatc

pdeD.A gatcacctgcaggatttatcaaacggcgtaactacg

pdeD.B gatcacggatccctgaaatttcatgagtcccatcatc

pdeD.C gtacacggatccggttatctggttaataagccgtttcc

pdeD.D tcgatagaattctgcgccatacagtttctcattacg

pssA.A: gggctgcagaatttgttgtaatttgtcgaca

pssA.B: ttacgcattccgctcaccggatttaactttccttatcat

pssA.C: ggtgagcggaatgcgtaa

pssA.D: gggggatccagaagcatctgtaattgcttt

pssB.A: gggctgcagaaactttgaaaaggcgacag

pssB.B: atatttcttcatgtaaagccgaatacttataatcatttttttacg

pssB.C: cggctttacatgaagaaatat

pssB.D: gggggatccgcctgtaatattatcggtatt

pssD.A: gggctgcagtaggcgcgttcgctttga

pssD.B: gctccggcgatatttacgcagccacattacagtaaattt

pssD.C: cgtaaatatcgccggagc

pssD.D: gggggatcctttcgtgttttcttcttgaag

pssC.A gatcacctgcagagaaactttccggtcgatattgcg

pssC.B cgtcacatacatcaacactggtatttcgggcacttttcg

pssC.C cccgaaataccagtgttgatgtatgtgacgtattgccagc

pssC.D tcgatagaattcgaccatctggcaaatcagtgtagac

pssE.A gatcacctgcagacaagattacttcatcagcatgc

pssE.B tcgataggatcctttcttcatttttatttctcctcacttcc

pssE.C gatcacggatccgacgtgtaaaatagagagttaccttc

pssE.D tcgatagaattcacagtaagtacagctctcacatc

Protein overexpression in pET23a

pdeB.F cactgaggatccgatatctcgagcacggaaatatg

pdeB.R cactgagtcgacatcaatggaatacttttgtttaatttcttcc

pdeC.F1 cactgaggatccaaaaagccctccgtacgtgag

pdeC.R cactgagtcgacatgaaaataataaccttgcgcatagggaatg

pdeD.F cactgaggatccggactcatgaaatttcagctttttattcaacc

pdeD.R cactgagtcgacaaccggaaacggcttattaaccag

Protein overexpression in pMAL-c2x

pdeC.F2 cactgaggatccatgaaaaagccctccgtacgtgag

pdeC.R2 cactgactgcagttaatgaaaataataaccttgcgcatagggaatg

pssE.F cactgaggatcctttacaagaaatacgtcaagacttcaagaag

pssE.R cactgactgcagttacacgtcatattctaattcttttgtcgc
